# Real-world effectiveness of medication-assisted treatment and psychotherapy for opioid use disorder: a national multi–health care organization analysis

**DOI:** 10.3389/fpsyt.2026.1741907

**Published:** 2026-04-28

**Authors:** Nicholas M. Graziane

**Affiliations:** Departments of Anesthesiology and Perioperative Medicine and Neuroscience and Experimental Therapeutics, Penn State College of Medicine, Hershey, PA, United States

**Keywords:** integrated care, medication for opioid use disorder (MOUD), opioid use disorder (OUD), psychotherapy, treatment retention

## Abstract

**Background:**

Harm reduction strategies for opioid use disorder (OUD) emphasize pragmatic, evidence-based approaches that reduce overdose risk, relapse, and other adverse outcomes without requiring abstinence. Medication for opioid use disorder (MOUD) and structured psychotherapy represent core harm-reduction modalities, yet their real-world comparative effectiveness, alone and in combination, remains underexplored at scale.

**Methods:**

A retrospective cohort study was conducted using the TriNetX Research Network, comprising de-identified electronic health records from 112 U.S. health systems. 18,047 adults aged 18–45 were identified with a diagnosis of opioid dependence (ICD-10 F11.20) between 2016 and 2025. Subjects were assigned to eight mutually exclusive treatment cohorts: no treatment (Cohort 1); buprenorphine alone (Cohort 2); methadone alone (Cohort 3); psychotherapy alone (30 minutes (Cohort 4), 45 minutes (Cohort 5), or 60 minutes (Cohort 6)); buprenorphine + psychotherapy (Cohort 7); and methadone + psychotherapy (Cohort 8), with combination treatments defined within a ±30-day window. Cox proportional hazards models estimated adjusted hazard ratios (aHRs) for remission (F11.21, F11.11) within 12 months.

**Results:**

Buprenorphine (aHR = 2.33; 95% CI: 1.85–2.94), methadone (aHR = 2.50; 95% CI: 2.05–3.04), and psychotherapy (30 min: aHR = 2.18; 45 min: aHR = 2.38) were each independently associated with significantly higher remission compared to no treatment. The combination of buprenorphine + psychotherapy yielded the strongest effect (aHR = 5.26; 95% CI: 2.68–10.32). Anxiety diagnoses and gabapentinoid prescriptions were positively associated with remission; benzodiazepine co-prescription was negatively associated.

**Conclusions:**

In this first national-scale, multi–health-care-organization analysis, both pharmacologic and psychosocial harm-reduction interventions were independently associated with improved OUD remission, with additive benefit when integrated. These findings underscore the value of embedding comprehensive, multimodal harm-reduction services within routine care and support policies promoting equitable access to both MOUD and behavioral health supports across diverse health systems.

## Introduction

Opioid use disorder (OUD) remains one of the most pressing public-health challenges in the United States, responsible for more than 80,000 overdose deaths annually and imposing profound social, medical, and economic burdens (1–3). Decades of clinical and epidemiologic research have established that OUD is a chronic, relapsing medical condition rather than a moral failing, yet stigma and policy restrictions continue to limit access to evidence-based treatment, including medication for opioid use disorder (MOUD), defined as the use of FDA-approved medications to reduce withdrawal, craving, and overdose risk, and psychosocial or behavioral therapies, which target the cognitive, emotional, and environmental factors that contribute to continued use (4–9). National data indicate that fewer than one-third of individuals with OUD receive MOUD, and fewer still engage in sustained behavioral therapy (1, 10–12).

MOUD, principally methadone, buprenorphine, and naltrexone, has shown to reduce opioid use, lower mortality, and improve retention in treatment (13–19). Buprenorphine and methadone act as opioid receptor agonists, partial and full, respectively, that mitigate withdrawal and craving, while extended-release naltrexone blocks the reinforcing effects of opioids. In parallel, psychosocial interventions such as cognitive behavioral therapy (CBT), motivational interviewing, contingency management, and emerging mindfulness-based approaches target the psychological, behavioral, and social drivers of relapse (20–24). When combined, pharmacologic and behavioral modalities can produce additive benefits for some individuals, improving treatment adherence and long-term recovery compared with medication or counseling alone (25–29). In the present study, harm reduction is conceptualized broadly to include both pharmacologic and psychosocial interventions that reduce opioid-related harms and support clinical improvement, including remission, without requiring strict abstinence. Within this framework, structured psychotherapy is considered a complementary modality that enhances behavioral regulation, treatment engagement, and risk reduction alongside MOUD.

While randomized controlled trials and regional cohort studies support the efficacy of MOUD and psychotherapy (30, 31), most are limited by small sample sizes, short follow-up intervals, or homogeneous patient populations. Few analyses have evaluated whether treatment effectiveness varies across demographic and clinical characteristics such as sex, age, psychiatric comorbidity, or chronic-pain status. Moreover, existing real-world studies typically derive from single health-system or state-level data and therefore cannot capture the heterogeneity of practice patterns across the United States. To date, few studies have leveraged a nationwide, multi-institutional electronic health record (EHR) databases to examine how demographic and clinical covariates influence the real-world effectiveness of MOUD and psychotherapy in OUD.

To address this critical gap, the present study examines the association between MOUD, psychotherapy, and their combination on remission outcomes in individuals with opioid dependence using a large, multi-institutional electronic health record database. By examining treatment effects across sex, age, and psychiatric comorbidities within a large, diverse population, this study provides the first national-scale, real-world assessment of OUD treatment effectiveness and retention across demographic strata.

## Methods

### Data source

A retrospective, observational cohort study was conducted using the TriNetX Research Network, a federated EHR database aggregating de-identified clinical data from 112 U.S. health-care organizations. Individuals aged 18–45 years with a diagnosis of opioid dependence, uncomplicated (ICD-10 F11.20) between January 1, 2016 and October 31, 2025 were included. Individuals included in the study were aged 18–45 to focus on a population where the onset and progression of OUD is most common and to reduce age-related heterogeneity in treatment patterns, comorbidities, and remission likelihood. Age 45 was selected as an upper bound to capture early- to mid-adulthood, where treatment access and retention dynamics differ from older adults with long-standing substance use histories or age-related medical complexity (32–34).

### Cohorts

Cohorts were defined independently within the TriNetX network based on a documented ICD-10 diagnosis of opioid dependence (F11.20) and the presence of at least one qualifying treatment encounter. For single-modality groups (MOUD or psychotherapy), individuals were excluded if they had records of other qualifying treatment modalities during the study period. For combined treatment cohorts, participants were included only if they had evidence of both MOUD and psychotherapy within a ±30-day window, selected to reflect real-world temporal clustering of coordinated care, without documentation of additional modalities. The index date was defined as the earliest encounter within the qualifying treatment window. This approach prioritized treatment specificity and temporal alignment while minimizing misclassification due to scattered or ambiguous treatment history. Treatment cohorts were defined to preserve clinically meaningful distinctions between pharmacologic agents and psychotherapy intensity. This stratified approach was selected to capture heterogeneity in real-world treatment exposure and avoid obscuring potential differences in effectiveness across modalities. Sensitivity analyses were conducted using collapsed treatment categories (e.g., MOUD and psychotherapy) to assess the robustness of the primary analyses. Naltrexone, although an FDA-approved medication for opioid use disorder, was not included as a primary cohort due to differences in clinical use and limitations in EHR-based measurement, including inability to reliably distinguish formulation type (oral vs extended-release) and assess adherence, which may affect interpretability of outcomes.

Cohort 1 - No MOUD or psychotherapy (PsyTx) (Control): Patients with F11.20 (opioid dependence, uncomplicated) and no record of MOUD-related procedure or drug codes (1819, 6813, 7242) or psychotherapy CPT (Current Procedural Terminology) codes (90832–90838). Exclusions included palliative care (Z51.5), pregnancy (O10–O9A), and personal history of malignancy or chronic disease (Z85–Z87).

Cohorts 2 - 3 - MOUD: Patients with F11.20 who received at least one medication used in the treatment of opioid use disorder, identified by CPT/HCPCS (Healthcare Common Procedure Coding System) procedure or National Drug Codes: Buprenorphine (1819) (Cohort 2) or Methadone (6813) (Cohort 3).

### Psychotherapy and combined-treatment sub-cohorts

To assess behavioral interventions, additional exposure groups were constructed based on psychotherapy billing codes:

Cohorts 4-6 - Psychotherapy Only: Encounters including any of the following CPT codes without MOUD: 90832/90833 (30 min) (Cohort 4), 90834/90836 (45 min) (Cohort 5), 90837/90838 (60 min) (Cohort 6).

Cohorts 7-8 - MOUD + Psychotherapy Combination: Encounters meeting criteria for MOUD (buprenorphine (Cohort 7) or Methadone (Cohort 8)) with at least one psychotherapy CPT code occurring within 1 month of the MOUD encounter (± 30 days). This time frame was chosen to balance specificity and inclusivity: a narrower window (e.g., 7–14 days) would likely miss valid integrated care encounters due to variability in scheduling, billing, or documentation lag across health systems. A broader window (e.g., 60–90 days) risks capturing unrelated, temporally distinct treatment episodes. The 30-day interval thus reflects a pragmatic compromise informed by prior EHR-based addiction studies that used similar thresholds to operationalize multimodal care (35–38).

### Exclusion criteria

To minimize clinical heterogeneity unrelated to treatment course, encounters were excluded if any of the following ICD-10 codes were present at or prior to the index event: Individuals were excluded with documented palliative care (Z51.5), active pregnancy (O10–O9A), or personal history of malignancy and complex chronic disease (Z85–Z87) to minimize clinical heterogeneity and avoid confounding due to care priorities unrelated to OUD remission. These exclusions enhanced internal validity and statistical stability across cohorts while acknowledging that this may limit generalizability to older or medically complex patients.

### Outcomes

Primary outcome: opioid dependence or abuse in remission (ICD-10 F11.21, F11.11) within 1-year after the index event. Index date was defined as the first qualifying encounter meeting inclusion criteria. A 12-month follow-up period was selected because retention in medication for opioid use disorder commonly declines over the first year of treatment, making this interval clinically meaningful for assessing treatment response and durability of engagement (39–41). Remission codes (F11.21, F11.11) were selected as the primary outcome because they offer a consistent, structured representation of clinical improvement that is broadly recognized in EHR-based research. While these codes do not directly measure abstinence, retention, or relapse, they reflect a clinician-documented assessment of meaningful progress in OUD recovery. Prior validation of behavioral health coding has shown that remission codes, though variably used, tend to be applied in cases of sustained stability or treatment response (Keller, 2003). TriNetX does not support reliable identification of relapse events, ongoing opioid use, or structured retention endpoints due to gaps in prescription continuity and variability in behavioral health documentation. Therefore, remission was used as the most consistently available and clinically relevant outcome for real-world comparative effectiveness analysis.

### Covariates

Models were adjusted for sex, age at index, and comorbid psychiatric or medical conditions (F32, F33, F41, F41.3, F10–F19, M54, G89, G47, B18.2, B20, Z72.0) as well as gabapentinoid (N02BF) and benzodiazepine derivative (CN302) prescriptions. Covariates were selected based on prior literature demonstrating their relevance to treatment outcomes in OUD. Psychiatric diagnoses (e.g., depression, anxiety, other substance use disorders) are associated with lower retention and higher relapse rates in OUD populations (Iqbal et al., 2019; Seaberg et al., 2025; Tran et al., 2025). Pain and sleep disorders, as well as chronic infectious conditions like hepatitis C and HIV, represent common comorbidities that may influence treatment selection or prognosis (NIDA, 2020). Gabapentinoids and benzodiazepines were included due to their frequent co-prescription in OUD care and their known risks for misuse or drug interactions (42–45).

### Statistical analysis

Cox proportional hazards regression models were used to estimate adjusted hazard ratios (aHRs) and 95% confidence intervals (CIs) for remission. Cox proportional hazards models do not require equal group sizes and can accommodate imbalanced cohorts through likelihood-based estimation. However, smaller cohorts may yield less precise estimates, reflected in wider confidence intervals (46–50). Analyses were conducted within the TriNetX analytics environment using de-identified, real-world data. TriNetX performs Cox regression on aggregated data without access to individual-level identifiers. Statistical significance was set at *p* < 0.05.

## Results

### Participant characteristics

A total of 18,047 individuals aged 18–45 years with a diagnosis of opioid dependence (F11.20) met inclusion criteria across seven treatment cohorts (**Table 1**). The no-treatment group was the largest (n = 11,066), followed by methadone (n = 2,334) and buprenorphine (n = 1,296). Psychotherapy-only cohorts were substantially smaller than medication groups, ranging from 97 participants (45 min) to 224 (30 min). Combined medication + psychotherapy groups were limited (n = 37–76), reflecting sparse co-documentation of integrated care encounters within real-world electronic health records.

**Table 1:** Baseline demographic characteristics of study participants by treatment cohort.

	No treatment (control) (11,066)	Bupr. (1,296)	Metha. (2,334)	PsyTx 30 min (224)	PsyTx 45 min (97)	PsyTx 60 min (165)	Bupr. + PsyTx (45)	Metha. + PsyTx (76)
Age at index (mean ± SD)	30.8 ± 5.81	32.1 ± 5.71	32.8 ± 5.55	31.9 ± 6.43	30.8 ± 6.38	28.8 ± 6.73	29.8 ± 6.61	30.9 ± 5.54
Sex (M/F)	6,839/4,227	751/555	1,309/1,025	137/87	57/40	110/55	22/21	46/27
Ethnicity (Not Hispanic or Latino/Hispanic or Latino)	7,771/2,177	859/350	1,571/372	193/17	85/10	147/15	41/10	65/10

Bupr, buprenorphine; Metha, methadone; PsyTx, psychotherapy; Numbers in parenthesis under groups represent total subject counts.

The mean age at index (mean ± SD) ranged from 28.8 ± 6.7 years (psychotherapy 60 min) to 32.8 ± 5.6 years (methadone) (**Table 1**). Across all cohorts, the majority of participants were male (59–67%), consistent with national OUD treatment demographics (1, 51–54).

Ethnicity data were incomplete for a subset of encounters; among cases with documented ethnicity, most were non-Hispanic (approximately 75–85% across cohorts) (**Table 1**). Regional distribution of patients by treatment cohort is summarized in **Supplementary Table 1**. Although TriNetX does not provide site-identifiable geographic information for participating health systems, patient-level regional representation was observed across all major U.S. Census regions, with the greatest concentration in the Northeast and Midwest.

### Remission outcomes

**Figure 1:**
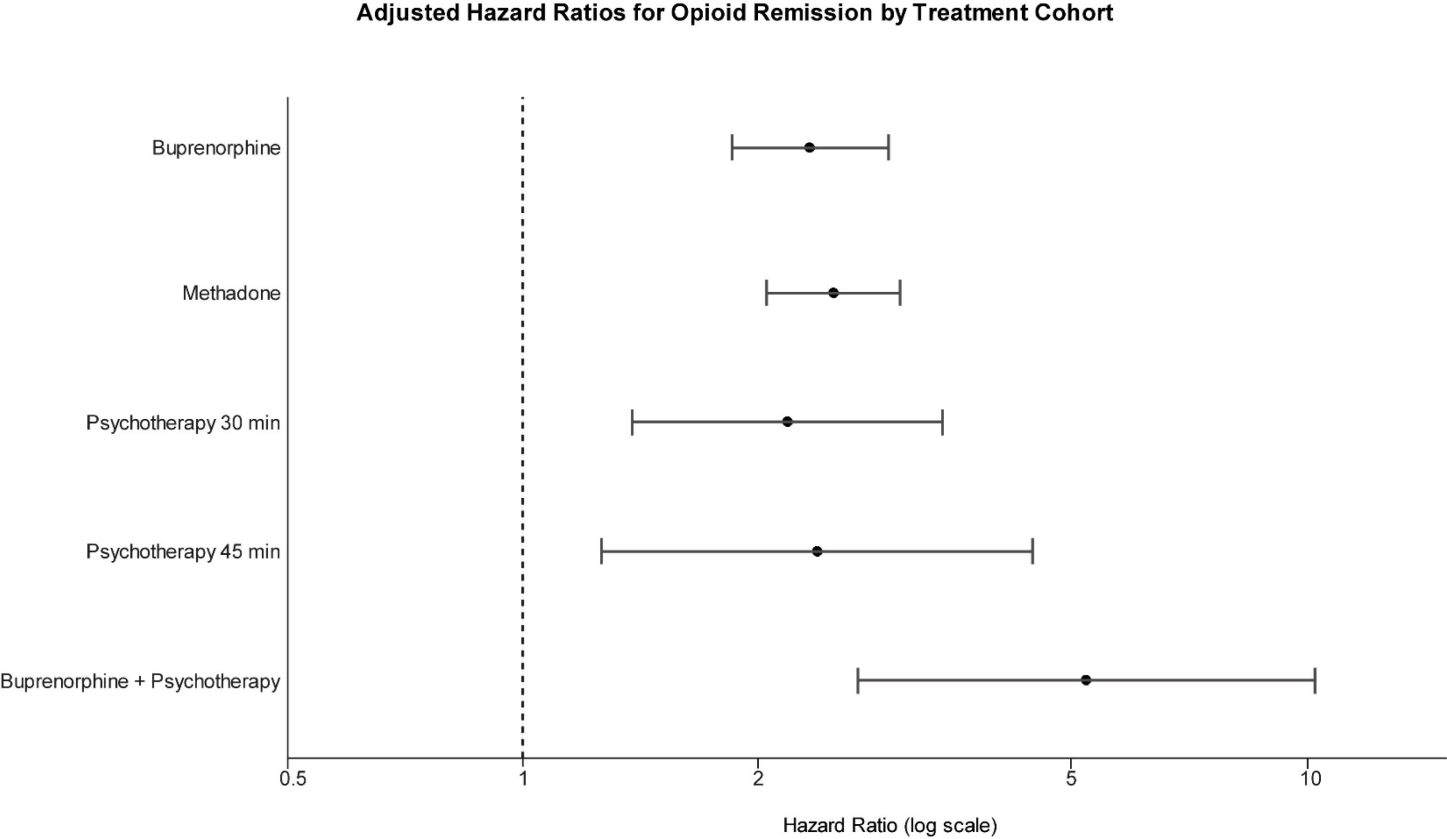
Forest plot of adjusted hazard ratios (aHRs) for 1-year remission outcomes by treatment cohort. HRs > 1 indicate greater remission hazard relative to no treatment; horizontal lines denote 95% confidence intervals.

Across seven treatment cohorts, buprenorphine, methadone, and structured psychotherapy were each independently associated with a higher likelihood of remission relative to no treatment (**Table 2**; **Figure 1**). Sensitivity analyses using collapsed treatment categories yielded consistent findings, with both MOUD (aHR = 2.31, 95% CI 1.96–2.73) and psychotherapy (aHR = 3.17, 95% CI 2.35–4.27) remaining significantly associated with remission (**Supplementary Table 3**).

**Table 2:** Hazard ratios (HR) for opioid remission by treatment cohort.

Cohort (cohort 1 vs no treatment)	Primary treatment exposure (ICD-10 code)	Outcome – remission HR (95% CI)	*P* value	Significant covariates (*p* < 0.05) (ICD-10 code, covariate, directionality of remission, hazard ratio)	Interpretation/key observation
1	Buprenorphine (1819)	2.33 (1.85–2.94)	< 0.0001	F41 Anxiety ↑ HR 1.62; N02BF Gabapentinoids ↑ HR 1.57; CN302 Benzodiazepines ↓ HR 0.59	Effective for remission; benzodiazepine co-use reduces benefit
2	Methadone (6813)	2.50 (2.05–3.04)	< 0.0001	F41 ↑ HR 1.47; B18.2 Hep C ↓ HR 0.59; CN302 ↓ HR 0.51	Strong remission signal; Hep C and benzos predict worse outcomes
3	Psychotherapy 30 min (90832)	2.18 (1.38–3.44)	0.0009	F41 ↑ HR 1.55; N02BF ↑ HR 1.60; CN302 ↓ HR 0.60	Behavioral therapy alone beneficial
4	Psychotherapy 45 min (90834)	2.38 (1.26–4.49)	0.0076	F41 ↑ HR 1.60; N02BF ↑ HR 1.61; CN302 ↓ HR 0.62	Reinforces effect of psychotherapy dose
5	Buprenorphine + Psychotherapy	5.26 (2.68–10.32)	< 0.0001	F41 ↑ HR 1.60; N02BF ↑ HR 1.57; CN302 ↓ HR 0.60	Strongest effect observed
6	Methadone + Psychotherapy	Model error (NaN)	–	–	Small N or collinearity
7	Psychotherapy 60 min (90837–90838)	Model error (NaN)	–	–	Insufficient sample for stable model

Hazard ratios (HRs) represent the relative likelihood of achieving remission (F11.21, F11.11). HR > 1 indicates an increased probability of remission; HR < 1 indicates reduced likelihood of remission. “Significant Covariates” denote variables with *p* < 0.05 in adjusted Cox models. Arrows indicate directionality relative to remission likelihood (↑ = higher remission probability; ↓ = lower remission probability). Model error (Not a Number (NaN)): Model failed to converge due to sparse events, small sample size, or high collinearity. These estimates are omitted to prevent misleading inference.

After adjustment for sex, age, and comorbid psychiatric and medical conditions, strong associations with remission were observed for methadone (aHR = 2.50, 95% CI 2.05–3.04, *p* < 0.0001) and buprenorphine (aHR = 2.33, 95% CI 1.85–2.94, *p* < 0.0001). Structured psychotherapy alone was also associated with increased remission likelihood (PsyTx 30 min: aHR = 2.18, 95% CI 1.38–3.44, *p* = 0.0009; PsyTx 45 min: aHR = 2.38, 95% CI 1.26–4.49, *p* = 0.0076) (**Table 2**; **Figure 1**).

The combination of buprenorphine + psychotherapy produced the largest observed effect (aHR = 5.26, 95% CI 2.68–10.32, *p* < 0.0001), suggesting an added benefit of pharmacologic stabilization coupled with behavioral therapy. Models for methadone + psychotherapy and psychotherapy (60 min) failed to converge, likely due to small sample sizes and limited event counts. Additionally, estimates for combined treatment groups and psychotherapy-only cohorts should be interpreted cautiously due to small sample sizes and wider confidence intervals, particularly for the buprenorphine + psychotherapy cohort.

**Table 2** summarizes all cohort comparisons, including significant covariates for anxiety disorders (F41), gabapentinoid prescriptions (N02BF), and benzodiazepine co-use (CN302). Across several models, anxiety and gabapentinoid prescriptions were associated with a greater likelihood of remission (HR > 1.5), whereas concurrent benzodiazepine use was associated with a lower likelihood of remission (HR < 0.6) (**Supplementary Table 2**).

## Discussion

This large, national, real-world analysis spanning 112 U.S. health-care organizations demonstrates that both MOUD and structured psychotherapy are strongly associated with remission from opioid dependence. After covariate adjustment, buprenorphine and methadone treatment more than doubled the likelihood of remission (aHR ≈ 2.3–2.5), while psychotherapy independently conferred a comparable benefit. The combination of pharmacologic and behavioral treatment produced the greatest effect, with a more than fivefold increase in remission relative to untreated individuals.

Our results are consistent with randomized and observational studies that demonstrate agonist-based therapy improves retention, reduces illicit opioid use, and lowers mortality (15, 55–60). Methadone’s full μ-opioid agonism and buprenorphine’s partial agonism provide sustained receptor occupancy, mitigating withdrawal and craving (61, 62). These mechanisms underlie the superior outcomes relative to detoxification or abstinence-based approaches (63, 64). By demonstrating equivalent or stronger effects in a broad, heterogeneous EHR sample, our findings underscore that these benefits generalize to real-world community and academic settings rather than being confined to specialized research cohorts.

Psychotherapy, particularly cognitive behavioral therapy, motivational interviewing, and contingency management, has repeatedly been shown to reduce relapse risk and improve coping, mood regulation, and treatment adherence among individuals with OUD (65, 66). In our analysis, both 30- and 45-minute psychotherapy sessions were independently associated with remission, with hazard ratios similar to pharmacotherapy alone. Our findings complement evidence from emerging mindfulness- and body-based interventions, such as Mindfulness-Oriented Recovery Enhancement (MORE) (67), Tai Chi Easy (68), and Rhemercise (69), which suggest that behavioral strategies targeting emotion regulation and craving may further enhance outcomes when integrated with MOUD (70). These converging data reinforce the clinical value of psychotherapy as an essential component of OUD care rather than an optional adjunct.

The most striking observation in our cohort was the added benefit of combined MOUD and psychotherapy, yielding a fivefold higher likelihood of remission relative to no treatment. This added benefit has been hypothesized but rarely quantified in large populations. However, this conclusion is primarily based on the buprenorphine + psychotherapy cohort, a small subgroup (n = 45) with wide confidence intervals, and should therefore be interpreted cautiously. Other combined groups could not be modeled due to sparse events. Prior research provides conflicting evidence on whether adding counseling or CBT provides additive benefits to pharmacotherapy for OUD, and some studies indicate that while both pharmacotherapy alone and combined treatment are effective, adding further therapy does not yield significant additional benefits for opioid use outcomes (28, 29, 71–73). Conversely, other studies suggest that adding behavioral therapy can improve outcomes, particularly when patients have co-occurring psychiatric disorders (74, 75). Our findings are consistent with this mixed literature, suggesting that combined approaches may confer benefit in specific patient subgroups, but that the magnitude and generalizability of this effect remain uncertain.

### Consistency across sex, age, and psychiatric comorbidity

The protective associations of MOUD and psychotherapy remained statistically significant after adjusting for sex, age, and psychiatric or pain-related comorbidities, suggesting these factors did not fully account for treatment effects. However, because subgroup or interaction analyses were not conducted, it is not possible to determine whether these associations were consistent across demographic or clinical subgroups. While reports have highlighted important demographic disparities in OUD remission (76–78), our findings indicate that these effects do not negate the overall benefit of treatment, supporting that, although baseline risks differ, MOUD and psychotherapy retain effectiveness across demographic strata.

In adjusted models, comorbid anxiety disorders (F41) and gabapentinoid prescriptions (N02BF) were independently associated with a higher likelihood of remission, suggesting that patients with these clinical features may be more closely engaged with ongoing treatment or benefit from enhanced monitoring and supportive care. Conversely, concurrent benzodiazepine prescriptions (CN302) were associated with a lower likelihood of remission. This finding may reflect either pharmacologic interference or differences in patient complexity and prescribing patterns rather than a direct causal relationship. These covariate effects underscore the need for comprehensive, coordinated care that addresses psychiatric comorbidities and optimizes co-prescribing practices within OUD treatment programs.

While several covariates demonstrated consistent associations across models, such as anxiety disorders and gabapentinoid prescriptions correlating with higher remission rates, and benzodiazepine use associated with lower remission, these findings should be interpreted cautiously. These patterns may reflect real differences in treatment trajectories or symptom presentation, but they could also arise from unmeasured confounding. For instance, patients prescribed benzodiazepines may have more severe psychiatric comorbidity, higher baseline relapse risk, or different provider monitoring practices that influence remission coding. Similarly, gabapentinoid use may signal more complex pain presentations rather than a direct facilitator of remission. As this study was not designed to establish causal pathways for covariates, these associations should be viewed as exploratory and hypothesis-generating rather than conclusive.

Digital and telehealth-based psychosocial interventions have gained attention for expanding access and engagement in OUD care. Telehealth delivery can reduce common barriers to treatment, including transportation, geographic limitations, scheduling constraints, and stigma, thereby increasing accessibility to counseling services, particularly in rural or underserved populations. Studies of web- and smartphone-delivered CBT, virtual mindfulness, and tele-counseling have demonstrated feasibility and modest improvements in retention and craving reduction (79–82). While our EHR dataset did not isolate digital delivery modes, the observed equivalence of psychotherapy effects across settings supports the growing evidence that behavioral engagement, whether in-person or remote, is a key determinant of success. Integration of telehealth counseling within MOUD programs could therefore represent an effective scalability strategy.

### Limitations

Several limitations warrant consideration: First, as a retrospective observational analysis, causality cannot be inferred; unmeasured confounders (e.g., motivation, social support, treatment readiness) may influence both treatment selection and outcomes. Second, remission was defined based on ICD-10 codes (F11.21 or F11.11) within one year of index treatment. While this approach offers a standardized, reproducible EHR-based metric, it reflects clinician documentation rather than direct behavioral measures such as abstinence, continued use, or relapse. Relapse events may not be consistently captured in structured EHR data and may be underdocumented, particularly if care occurs outside participating health systems. As a result, remission status may not fully reflect longitudinal recovery trajectories, and patients who relapse after initial remission or experience intermittent use may still be classified as remitted. Thus, observed associations reflect documented clinical status within the EHR rather than definitive or sustained recovery outcomes.

Third, psychotherapy exposure was identified using CPT billing codes and may not reflect session content, fidelity, or intensity. The ±30-day window for defining combined MOUD and psychotherapy was selected based on EHR conventions used in prior research, but it may not fully distinguish coordinated, integrated care from temporally overlapping or coincidental treatment exposure, and may also fail to capture all valid instances of integrated care in settings with asynchronous or fragmented documentation. Fourth, the models adjusted for key clinical covariates available in TriNetX (e.g., sex, age, psychiatric/pain diagnoses, gabapentinoids, benzodiazepines), but important determinants such as ethnicity, socioeconomic status, housing instability, neighborhood-level deprivation (e.g., Area Deprivation Index), insurance type, and justice system involvement were not available. As such, residual confounding is possible, particularly with respect to disparities in access or engagement. Fifth, there is a risk of unmeasured confounding related to treatment adherence and indication. For example, individuals who engage in MOUD or psychotherapy may differ systematically in motivation, health literacy, or social support (“healthy adherer” bias), which could independently influence outcomes. Similarly, clinicians may preferentially prescribe buprenorphine to patients perceived as more stable or lower risk, introducing confounding by indication. While our models adjusted for psychiatric and medical comorbidities, these proxies may not fully account for such selection effects.

Sixth, although the analysis period extended through October 2025, the one-year outcome requirement means that patients indexed late in the window may not have had complete follow-up. Incomplete follow-up may limit the ability to detect relapse events within the observation period, further contributing to potential misclassification of remission status. Longer follow-up periods (e.g., beyond 12 months) may provide additional insight into sustained remission and relapse risk over time. Seventh, although overall sample size was large, several subgroups, particularly the psychotherapy-only and combined MOUD + psychotherapy cohorts, were small (n = 37–76), which limited model stability. Models for methadone + psychotherapy and 60-minute psychotherapy failed to converge or yielded unstable estimates, preventing full evaluation of treatment synergy in these groups. Even in the buprenorphine + psychotherapy group (n = 45), while the association with remission was statistically significant, the wide confidence interval suggests imprecision and possible overestimation. These results should be interpreted with caution, and future work with larger samples should confirm whether these effects generalize.

Eighth, comparisons were made against a single pooled no-treatment group (n = 11,066). While this increases statistical power and facilitates cross-cohort comparisons, it assumes a uniform risk profile across all reference observations. Differences in time-at-risk or cohort-specific confounding could subtly bias results. Ninth, models were adjusted for key demographic and clinical covariates, but the analysis was limited to estimation of main effects and did not formally evaluate interaction terms or effect modification. As such, it remains unclear whether treatment effectiveness varies across specific subgroups (e.g., by sex, age, or comorbidity), and future studies should examine these interactions to better characterize differential treatment response. Finally, TriNetX data are derived from participating health systems, which may overrepresent large academic or urban institutions and underrepresent uninsured, rural, or non-academic populations. The geographic distribution of contributing health-care organizations within TriNetX is not available, and regional categorizations are platform-defined and not fully harmonized across cohorts, limiting direct comparability. Regional distributions therefore reflect patient-level data and may not correspond to the distribution of contributing health systems. As such, the generalizability of these findings to underserved or resource-limited settings may be limited. Additionally, the cohort definition spanned 2016–2025, encompassing the COVID-19 pandemic, during which documentation patterns and care delivery (e.g., via telehealth) may have changed. Variability in coding practices across institutions, particularly for behavioral outcomes such as remission and psychotherapy, could introduce bias.

## Conclusions

This study represents the first national-scale, multi–health-care-organization analysis of harm-reduction interventions for OUD using real-world electronic health record data. Both pharmacologic (methadone, buprenorphine) and psychosocial (structured psychotherapy) modalities were independently associated with increased likelihood of remission, and their combination conferred the greatest benefit. These findings extend evidence from controlled trials to large, heterogeneous healthcare populations, demonstrating that pharmacologic stabilization and behavioral engagement remain complementary strategies within harm-reduction–oriented care.

By showing that integrated MOUD and psychotherapy are effective across diverse demographic and clinical contexts, this study reinforces the central tenet of harm reduction, that pragmatic, accessible, and patient-centered approaches can substantially reduce the burden of OUD even outside abstinence-based frameworks. Real-world data networks such as TriNetX provide a critical infrastructure for evaluating and scaling such approaches, offering population-level insight into how multimodal care models function across health systems.

In practice, implementation of integrated MOUD and psychotherapy may be most feasible in settings with established behavioral health infrastructure, including integrated primary care clinics, specialty addiction treatment programs, and health systems with embedded mental health services. However, real-world barriers such as limited access to trained behavioral health providers, fragmented care delivery, reimbursement constraints, and geographic disparities, particularly in rural or underserved regions, may limit widespread adoption of multimodal care.

These results underscore the need for equitable access to both MOUD and behavioral health supports within routine clinical practice and for policy frameworks that embed comprehensive harm-reduction services into mainstream healthcare. Future work should examine how digital and telehealth-delivered psychosocial interventions can further extend the reach of evidence-based harm reduction to underserved and high-risk populations.

## Data Availability

The original contributions presented in the study are included in the article/**Supplementary Material**. Further inquiries can be directed to the corresponding author.

## Supplementary material

Supplementary Table 1 Treatment Uptake by U.S. Region Across Cohorts (TriNetX Output). TriNetX was unable to provide geographical distribution for the Methadone + PsyTx cohort (100% of the cohort was from the United States).

Supplementary Table 2 Cox proportional hazards model results for remission (reference group = no treatment). Cox proportional hazards regression models estimate adjusted hazard ratios (aHRs) for remission (ICD-10 F11.21, F11.11) within 12 months after index treatment. Reference group = no MOUD or psychotherapy. Significant associations (p < 0.05) are shown in bold.

Supplementary Table 3 Sensitivity analysis of remission outcomes using collapsed treatment categories.
