# Supplementary material for: Real-world effectiveness of medication-assisted treatment and psychotherapy for opioid use disorder: a national multi–health care organization analysis
Source: Front Psychiatry. 2026 Apr 28;17:1741907. doi: 10.3389/fpsyt.2026.1741907 (PMC13161071; doi:10.3389/fpsyt.2026.1741907)
Supplement: Supplementary Table 1 — Treatment Uptake by U.S. Region Across Cohorts (TriNetX Output). TriNetX was unable to provide geographical distribution for the Methadone + PsyTx cohort (100% of the cohort was from the United States). [file SupplementaryFile1.pdf]

Supplementary Table S1. Regional Distribution by Treatment Cohort Across the U.S.

Cohort	Northeast (%)	Midwest (%)	South (%)	West (%)
No Treatment	53	22	5	20
Buprenorphine	51	33	3	13
Methadone	68	9	4	19
	**East (%)**		**West (%)**	
PsyTx 30 min	71		29	
PsyTx 45 min	81		19	
PsyTx 60 min	86		14	
Buprenorphine + PsyTx	68		32	
Methadone + PsyTx	-		-	

TriNetX was unable to provide geographical distribution for the Methadone + PsyTx cohort (100% of the cohort was from the United States).

Supplementary Table S2. Cox proportional hazards model results for remission (reference group = no treatment)

Covariate	Buprenorphine HR (95% CI)	Methadone HR (95% CI)	Psychotherapy 30 min HR (95% CI)	Psychotherapy 45 min HR (95% CI)	Buprenorphine + Psychotherapy HR (95% CI)
**Primary exposure (cohort)**	**2.33 (1.85–2.94)**	**2.50 (2.05–3.04)**	**2.18 (1.38–3.44)**	**2.38 (1.26–4.49)**	**5.26 (2.68–10.32)**
**Sex (Male)**	1.12 (0.93–1.35)	1.08 (0.91–1.29)	1.07 (0.87–1.32)	1.07 (0.87–1.33)	1.07 (0.87–1.33)
**Age at index**	1.00 (0.98–1.01)	0.99 (0.98–1.01)	0.99 (0.98–1.01)	0.99 (0.98–1.01)	0.99 (0.97–1.01)
**F32 Depressive episode**	1.09 (0.85–1.40)	1.06 (0.83–1.37)	1.19 (0.90–1.57)	1.20 (0.91–1.58)	1.25 (0.94–1.65)
**F33 Major depressive disorder, recurrent**	1.31 (0.94–1.81)	1.01 (0.70–1.44)	0.92 (0.62–1.36)	1.04 (0.71–1.52)	0.94 (0.63–1.40)
**F41 Other anxiety disorders**	**1.62 (1.30–2.02)**	**1.47 (1.19–1.83)**	**1.55 (1.21–1.98)**	**1.60 (1.25–2.05)**	**1.60 (1.25–2.05)**
**F41.3 Mixed anxiety disorders**	5.15 (0.72–37.0)	0 (0, 0)	0 (0, 0)	0 (0, 0)	0 (0, 0)
**F10 Alcohol-related disorders**	1.20 (0.94–1.54)	1.25 (0.99–1.59)	1.23 (0.95–1.60)	1.18 (0.91–1.55)	1.23 (0.94–1.61)
**F10–F19 Other substance use disorders**	2.40 (0.59–9.77)	4.62 (0.65–33.1)	1.28×10^{6} (0, 0)	8.78×10^{5} (0, 0)	2.40×10^{6} (0, 0)
**M54 Dorsalgia**	0.94 (0.65–1.36)	0.75 (0.52–1.09)	0.82 (0.53–1.26)	0.82 (0.53–1.26)	0.79 (0.51–1.22)
**G89 Pain, not elsewhere classified**	0.73 (0.47–1.11)	0.68 (0.45–1.02)	0.72 (0.45–1.16)	0.70 (0.43–1.13)	0.72 (0.44–1.16)
**G47 Sleep disorders**	1.05 (0.72–1.53)	1.18 (0.83–1.70)	1.01 (0.67–1.54)	1.05 (0.69–1.60)	1.03 (0.68–1.57)
**B18.2 Chronic hepatitis C**	0.96 (0.61–1.50)	**0.59 (0.36–0.96)**	0.77 (0.44–1.34)	0.85 (0.50–1.45)	0.72 (0.41–1.29)
**B20 HIV disease**	0.69 (0.17–2.78)	1.00 (0.32–3.11)	0.87 (0.21–3.50)	0.82 (0.20–3.33)	0.87 (0.22–3.54)
**Z72.0 Tobacco use**	1.25 (0.88–1.78)	1.28 (0.91–1.78)	1.12 (0.75–1.67)	1.30 (0.89–1.91)	1.22 (0.82–1.82)
**N02BF Gabapentinoids**	**1.57 (1.14–2.17)**	1.09 (0.77–1.54)	**1.60 (1.07–2.38)**	**1.61 (1.07–2.41)**	**1.57 (1.04–2.37)**
**CN302 Benzodiazepines**	**0.59 (0.45–0.79)**	**0.51 (0.39–0.67)**	**0.60 (0.42–0.83)**	**0.62 (0.44–0.87)**	**0.60 (0.42–0.84)**

Cox proportional hazards regression models estimate adjusted hazard ratios (aHRs) for remission (ICD-10 F11.21, F11.11) within 12 months after index treatment. Reference group = no MOUD or psychotherapy. Significant associations ($p < 0.05$) are shown in bold.

Supplementary Table S3. Sensitivity analysis of remission outcomes using collapsed treatment categories

Cohort (Cohort 1 vs No Treatment)	Primary Treatment Exposure (ICD-10 code)	Outcome – Remission HR (95 % CI)	*p* value	Significant Covariates (*p* < 0.05) (ICD-10 code, covariate, directionality of remission, hazard ratio)	Interpretation / Key Observation
**1**	Buprenorphine (1819) + Methadone (6813)	2.31 (1.96–2.73)	< 0.0001	F41 Anxiety ↑ HR 1.57; F10 Alcohol-related disorders ↑ HR 1.29; G47 Sleep disorders ↑ HR 1.58; G89 Pain ↓ HR 0.68; CN302 Benzodiazepines ↓ HR 0.58	Effective for remission; benzodiazepine co-use and pain are associated with reduced remission
**2**	Psychotherapy (30, 45, 60 minutes)	3.17 (2.35–4.27)	< 0.0001	F41 Anxiety ↑ HR 1.43	Effective for remission; anxiety is associated with increased remission.
